# Regulation of microglial TMEM119 and P2RY12 immunoreactivity in multiple sclerosis white and grey matter lesions is dependent on their inflammatory environment

**DOI:** 10.1186/s40478-019-0850-z

**Published:** 2019-12-11

**Authors:** Thecla A. van Wageningen, Eva Vlaar, Gijs Kooij, Cornelis A. M. Jongenelen, Jeroen J. G. Geurts, Anne-Marie van Dam

**Affiliations:** 1 Amsterdam UMC, Vrije Universiteit Amsterdam, Department of Anatomy and Neurosciences, Amsterdam Neuroscience, MS Center Amsterdam, Amsterdam, The Netherlands; 2 Present Address: Erasmus MC, Erasmus University Rotterdam, Center of lysosomal and metabolic diseases, Dept. Pediatrics and Clinical Genetics, Rotterdam, The Netherlands; 3 Amsterdam UMC, Vrije Universiteit Amsterdam, Dept. Molecular Cell Biology and Immunology, Amsterdam Neuroscience, MS Center Amsterdam, Amsterdam, The Netherlands; 4 Amsterdam UMC, location VUmc, Department of Anatomy and Neurosciences, De Boelelaan 1108, 1081 HZ Amsterdam, The Netherlands

**Keywords:** Multiple sclerosis, Homeostatic microglia, Cortical lesions, Demyelination, Subpial lesions

## Abstract

Multiple Sclerosis (MS) is the most common cause of acquired neurological disability in young adults, pathologically characterized by leukocyte infiltration of the central nervous system, demyelination of the white and grey matter, and subsequent axonal loss. Microglia are proposed to play a role in MS lesion formation, however previous literature has not been able to distinguish infiltrated macrophages from microglia. Therefore, in this study we utilize the microglia-specific, homeostatic markers TMEM119 and P2RY12 to characterize their immunoreactivity in MS grey matter lesions in comparison to white matter lesions. Furthermore, we assessed the immunological status of the white and grey matter lesions, as well as the responsivity of human white and grey matter derived microglia to inflammatory mediators. We are the first to show that white and grey matter lesions in post-mortem human material differ in their immunoreactivity for the homeostatic microglia-specific markers TMEM119 and P2RY12. In particular, whereas immunoreactivity for TMEM119 and P2RY12 is decreased in the center of WMLs, immunoreactivity for both markers is not altered in GMLs. Based on data from post-mortem human microglia cultures, treated with IL-4 or IFNγ+LPS and on  counts of CD3^+^ or CD20^+^ lymphocytes in lesions, we show that downregulation of TMEM119 and P2RY12  immunoreactivity in MS lesions corresponds with the presence of lymphocytes and lymphocyte-derived cytokines within the parenchyma but not in  the meninges. Furthermore, the presence of TMEM119^+^ and partly P2RY12^+^ microglia in pre-active lesions as well as in  the rim of active white and grey matter lesions, in addition to TMEM119^+^ and P2RY12^+^ rod-like microglia in subpial grey matter lesions suggest that blocking the entrance of lymphocytes into the CNS of MS patients may not interfere with all possible effects of TMEM119^+^ and P2RY12^+^ microglia in both white and grey matter MS lesions.

## Introduction

Multiple Sclerosis (MS) is the most common cause of acquired neurological disability in young adults. It is a chronic inflammatory, degenerative disease of the central nervous system (CNS), pathologically characterized by leukocyte infiltration of the CNS, demyelination of the white and grey matter, and subsequent axonal loss. From a clinical point of view, MS is very heterogeneous and is associated with an array of symptoms, including sensory and motor deficits, fatigue, cognitive and psychiatric disturbances [1, 2].

Microglia are considered to play an important role in MS lesion formation [3–7]. Dysfunction of the blood-brain-barrier leads to infiltration of leukocytes into the CNS, possibly attracted by antigens presented by microglia and/or by infiltrated macrophages [6, 8]. Indeed, activated, amoeboid-shaped microglia are present within active white matter lesions (WMLs) and in the rim of chronic-active WMLs, expressing MHC-II [9]. Pre-active lesions consisting of microglial nodules expressing MHC-II can also be found in the normal appearing white-matter, preceding demyelination and infiltration of leukocytes [10].

When studying the expression profile of microglia, at least two genes have been related to a homeostatic signature of microglia in the human and rodent brain, i.e. TMEM119 and P2RY12. Both TMEM119 and P2RY12 mRNA have been shown to be expressed only by microglia and not by infiltrating macrophages [11–14]. Interestingly, TMEM119 and P2RY12 immunoreactivity has been shown to be reduced in active WMLs compared to normal-appearing WM in post-mortem MS patient brain material which can indicate either a decrease in microglia presence in the WML or regulation of the microglia markers by the local inflammatory environment [15–17]. This last option is supported by observations that P2RY12 expression in human microglia is enhanced by the anti-inflammatory cytokine interleukin-4 (IL-4) [15, 18], whereas TMEM119 mRNA levels are reduced in mouse derived microglia treated with pro-inflammatory lipopolysaccharide in vitro [11], indicating that expression of both markers can be regulated by inflammatory cytokines.

Contrary to WMLs, to date, there has been no study on the expression of TMEM119 and P2RY12 in grey matter lesions (GMLs). However, recent studies utilizing single-cell RNA-seq have shown that microglia in normal appearing white matter (NAWM) and normal-appearing grey matter (NAGM) of MS patients differ in their gene expression pattern [19]. In line with this observation, it was already shown in normal rodent brain, that microglia derived from various brain regions show a region-specific expression profile [20, 21]. In that respect it is worth noting that, different from WMLs, microglia in MS GMLs only sparsely express MHC-II and show mostly a ramified or ‘reactive’ phenotype instead of an amoeboid, ‘active’ phenotype [22–24].

If we want to understand how microglia can contribute to MS lesion formation, more attention should be focused on microglia in GMLs. In GMLs, demyelination is as evident, or even more extensive [25–27] as in WMLs, but the microglial and inflammatory response appears different. Therefore, in order to expand the existing literature we identified and compared the expression of the homeostatic markers TMEM119 and P2RY12 in MS GMLs to WMLs. To this end, we used post-mortem human MS brain material containing subpial GMLs and various WML types, and leukocortical lesions to perform immunohistochemical analysis of TMEM119 and P2RY12. Moreover, the immunological status of the lesions was determined and the responsivity of human white matter (WM) and grey matter (GM) derived microglia to inflammatory mediators was assessed.

## Methods

### Post-mortem human brain tissue

Post-mortem brain material of MS patients was obtained from the Netherlands Brain Bank (NBB, Amsterdam, The Netherlands) and from the Biobank of the Amsterdam MS center (Amsterdam, The Netherlands). In compliance with all local ethical and legal guidelines, informed consent for brain autopsy and the use of brain tissue and clinical information for scientific research was given by either the donor or the next of kin. For immunohistochemical purposes, a total of 27 tissue blocks from 18 clinically diagnosed and pathologically verified MS patients were used. For isolating primary microglia, fresh NAWM and NAGM tissue was taken at autopsy from 12 patients with various neurological diseases. Clinicopathological information of patients from which brain material was used in this study, is provided in Table 1.

**Table 1:** Clinicopathological information of included patients for immunohistochemistry and primary microglia isolation

Patient	Age	Gender	Diagnosis	Disease duration * (years)	Post-mortem delay (h)	Cause of death	Lesions
*Immunohistochemistry*							
1	60	M	SPMS	16	8:49	Euthanasia	1 aWML, NAWM, NAGM
2	48	M	PPMS	18	6:35	Dehydration	2 aWML, 2 cWML, 2 sGML, NAWM, NAGM
3	66	F	PPMS	27	9:45	Pneumonia	1 sGML, NAWM, NAGM
4	52	F	PPMS	25	8:40	Euthanasia	1 cWML, 1 sGML, NAWM, NAGM,
5	74	F	PPMS	16	10:30	Respiratory failure	1 sGML, NAWM, NAGM,
6	65	F	SPMS	22	10:45	Brain infarction	2 cWML, 1 sGML, NAWM, NAGM,
7	66	F	SPMS	22	6:00	Unknown	2 aWML, 1 sGML, NAWM, NAGM
8	51	M	SPMS	20	11:00	Unknown	2 aWML, 1 cWML, NAGM
9	50	F	SPMS	12	9:05	Euthanasia	1 aWML, 1 sGML, NAWM,
10	50	M	SPMS	21	10:50	Euthanasia	1 aWML, NAWM, NAGM
11	54	M	PPMS	12	8:15	Euthanasia	1 cWML,1 sGML
12	54	F	SPMS	23	9:25	Respiratory failure	1 aWML
13	47	F	SPMS	27	8:35	Pneumonia	1 aGML
14	53	M	PPMS	2	5:30	Pneumonia	1 leukocortical lesion
15	41	F	SPMS	11	8:25	Natural causes	1 leukocortical lesion
16	45	M	SPMS	20	7:45	Cardiac Arrest	1 leukocortical lesion
17	54	F	SPMS	24	9:10	Dyspnea followed by palliative care	1 leukocortical lesion
18	57	F	SPMS	25	10:40	Euthanasia	1 leukocortical lesion
*Primary microglia isolation*							
15	81	M	PD	38	8:05	Septic Shock	
16	65	M	SPMS	34	9:30	Euthanasia	
17	51	F	SPMS	17	9:10	Euthanasia	
18	70	M	SPMS	33	9:25	Euthanasia	
19	81	F	PD	7	10:50	Respiratory Failure	
20	76	F	Hypokinesia / PD	9	9:15	Heart Failure	
21	67	F	PPMS	16	5:45	Euthanasia	
22	35	F	Neuropathic pain	8	5:20	Euthanasia	
23	65	F	MSA-P	3	7:05	Euthanasia	
24	67	M	PPMS	11	7:55	Euthanasia	
25	52	F	PPMS	2	9:30	Euthanasia	
26	83	F	PPMS	34	7:40	Ovarian Cancer	

*M* male, *F* female, *SP* Secondary progressive, *PP* Primary progressive, *PD* Parkinson’s Disease, *MSA-P* Multiple System Atrophy-Parkinsonism, * Starting from first diagnosis, *NAGM* normal appearing grey matter, *NAWM* normal appearing white matter, *cWML* chronic white matter lesion, *aWML* active white matter lesion, *sGML* subpial grey matter lesion, *aGML* active grey matter lesion

### Immunohistochemistry

After autopsy, dissected brain tissue was fixed in 4% formalin and subsequently embedded in paraffin. From the obtained cortical and subcortical tissue paraffin blocks, 10 μm sections were cut on a microtome and mounted on positively charged glass slides (Permafrost) and incubated on a heated plate for 1 h at 43 °C. Afterwards, slides were dried overnight in an incubator at 37 °C before being stored at room temperature (RT). Upon use for immunohistochemistry, tissue sections were heated to 58 °C for 30 min. Subsequently, sections were deparaffinized in xylene replacement (100%) and graded ethanol series (100, 96, 80 and 70%) to demi-water. For antigen retrieval, sections were heated to 90–95 °C in 10 mM Tris buffer containing 1 mM EDTA (Tris-EDTA, pH 9) or in 0.1 M citrate buffer (pH 6, see Table 2) for 30 min. in a conventional steam cooker. When cooled down to RT, sections were washed in TBS (pH 7.6) and incubated in TBS with 1% H2O2 for 20 min. to block endogenous peroxidase activity. Subsequently, after washes with TBS, the sections were incubated for 30 min. in TBS containing 0.5% Triton (TBS-T) and 5% milk powder (Campina, Zaltbommel, The Netherlands; block buffer) to block non-specific antibody binding.

**Table 2:** Primary antibodies used for immunohistochemistry

Primary antibody	Ab Dilution	Antigen Retrieval	Source (article number)
Rabbit anti TMEM119 C-terminus	1:500	Tris/EDTA pH 9	Atlas Antibodies, Sweden (HPA051870)
Rabbit anti Human P2Y12R C-terminus	1:200	Tris/EDTA pH 9	Anaspec, Netherlands (AS-55042A)
Mouse anti MHC-II (HLA-DR)	1:1000	Tris/EDTA pH 9	Clone LN3, Pierce, ThermoFisher (MA5–11966)
Rabbit anti Iba-1	1:1000	Citrate pH 6	WAKO Chemicals U.S.A. (019–19,741)
Mouse anti PLP	1:250	Tris/EDTA pH 9	Serotec (MCA839G)
Rabbit anti CD3	1:100	Citrate pH 6	DAKO, Denmark (A04520)
Mouse anti CD20	1:200	Tris/EDTA pH 9	DAKO, Denmark (M0755)
Mouse anti IL-4	1:500	Citrate pH 6	BioMatik (CAU29167)
Mouse anti IFN-γ	1:000	Tris/EDTA pH 9	Abcam, U.K. (ab218426)
Goat anti Iba-1	1:500	Tris/EDTA pH 9	Abcam, U.K. (ab5076)

*Ab* antibody

Primary antibodies were diluted in block buffer as indicated in Table 2, and the sections were incubated with the antibodies overnight at 4 °C. Then, sections were washed in TBS and incubated in block buffer containing corresponding biotinylated goat anti mouse IgGs (1:400, Jackson laboratories, Cambridge, UK) or biotinylated donkey anti rabbit IgGs (1:400, Jackson laboratories) at RT for 2 h. Subsequently, sections were washed in TBS and incubated for 1 h with horseradish peroxidase-labeled avidin-biotin complex (ABC complex, 1:400, Vector Labs) in TBS-T at RT. Finally, after washes in TBS and Tris-HCl, immunoreactivity was visualized by adding 3,3-diaminobenzidine (DAB, Sigma, St. Louis, USA) or NovaRED (Vector Labs, Peterborough, UK), and sections were counterstained with hematoxylin. Sections were subsequently dehydrated in graded series of ethanol, cleared in xylene and mounted with Entellan.

### Identification of multiple sclerosis lesion types

**Fig. 1:**
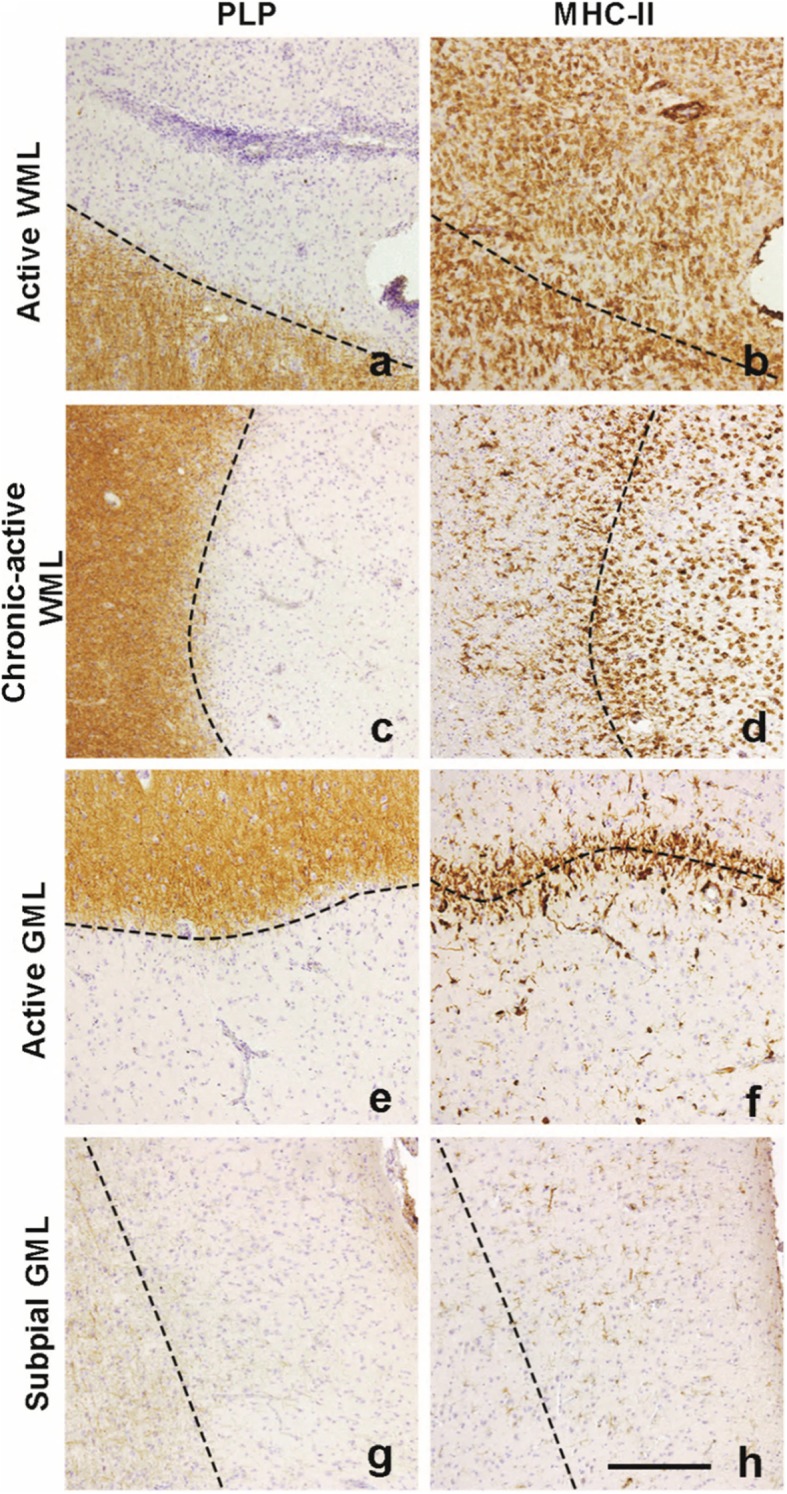
Representative images of lesion types used in this study. Lesions are characterized by loss of PLP staining and amount of MHC-II+ cells. A large amount of of MHC-II+ cells can be observed in the demyelinated area (**a**) in active WMLs (**b**). Chronic-active demyelinated WMLs (**c**) feature a ‘rim’ of MHC-II cells (**d**) which is also visible in demyelinated active GMLs (**e, f**). Subpial demyelinated (**g**) GMLs hardly show MHC-II+ cells (**h**). Scalebar (**a-h**) = 200 μm. Dashed lines indicate the edge of the lesion

MS lesion types were identified in post-mortem brain material by immunohistological staining for myelin proteolipid protein (PLP) and staining for the HLA-DR marker MHC-II. Lesion location was determined by the relative absence of PLP immunoreactivity indicating demyelinating/demyelinated areas. WML types were classified according to Kuhlmann et al. (2017) [9]. WML types were characterized as active when immunoreactivity for PLP was lost and a large number of amoeboid MHC-II^+^ cells was present in the demyelinating or demyelinated lesion (Fig. 1a, b). WMLs showing a ‘rim’ of MHC-II^+^cells around the demyelinated lesion with less, but still apparent amoeboid MHC-II^+^ cells in the center of the demyelinated lesion were deemed chronic-active WMLs (or mixed active/inactive according to Kuhlmann et al., (2017) (Fig. 1c, d). In contrast to WMLs, characterization of lesions in the GM is based on location rather than MHC-II^+^ activity [28]. Lesions showing loss of myelin (as indicated by reduced PLP staining) from the outer layer into the cortex were deemed subpial lesions [9, 29]. They often present with little MHC-II^+^ cells (Fig. 1g, h) [23]. Lesion activity in GMLs is defined by the presence or absence of a rim of activated microglia surrounding the lesion area as defined by Peterson et al., (2001) [30]. One case showed a subpial GML with a clear rim of activated MHC-II^+^ cells, this lesion was deemed an active subpial GML (Fig. 1e, f) [23]. Type 1 (leukocortical) lesions featured a chronic-active white-matter demyelinated area, characterized by a rim of MHC-II^+^ cells and a grey-matter demyelinated area with a comparatively low amount of MHC-II^+^ cells similar to subpial GMLs (Additional file 1: Figure S1) [9]. In addition, WM areas with MHC-II^+^ microglial nodules, but no demyelination and not in close proximity to blood vessels were deemed pre-active lesions [10].

### Double-labeling immunohistochemistry

Sections were pretreated as described for single labeling above. Sections were incubated overnight at 4 °C with both primary antibodies (either TMEM119, P2RY12 or Iba-1, for dilutions see Table 2) diluted in block buffer. Sections were washed in TBS and incubated with alkaline phosphatase ImmPRESS anti-Rabbit IgG polymer detection kit (Vectorlabs) for 30 min. at RT. Subsequently, slides were washed again and incubated for 2 h at RT with a biotinylated donkey anti goat IgG’s (Iba-1; 1:400, Jackson laboratories). Subsequently, sections were washed in TBS and incubated for 1 h at RT with horseradish peroxidase labeled avidin-biotin complex (ABC complex, 1:400 Vectashield). Afterwards, slides were washed in TBS and immunoreactivity of TMEM119 or P2RY12 was then visualized by adding Liquid Permanent Red (LPR, DAKO) and Iba-1 immunoreactivity was visualized using the Vector SG Peroxidase kit (Vectorlabs). Subsequently, sections were washed and dried on a heated plate at 37 °C before being cleared in xylene and mounted with Entellan.

### Separation of color signals from double-labeled sections

Pictures of double labeled sections were taken at wavelengths ranging from 480 nm to 680 nm at 60x magnification using the Nuance multispectral imaging system (PerkinElmer). LPR stained cells and Vector SG stained cells were separated based on their light emission which yields images similar to fluorescently labeled antibodies. Using the open source software ImageJ [31], compositions of the separated signals were then made to visualize co-localization.

### Semi-quantitative analysis of immunoreactivity

Immunoreactivity detected in active and chronic-active WMLs, NAWM, subpial GMLs and NAGM was analyzed using ImageJ. Per lesion, depending on the lesion size, 1–2 images were made at 20x magnification using a Leica DM5000B microscope. Per NAWM and NAGM area, 2 images were made. All images analyzed had a region of interest (ROI) of 622 × 466 μm. Within these ROIs, signals from DAB and hematoxylin were separated using the color deconvolution plug-in [32]. From the subsequently acquired DAB images without heamatoxylin signal, an auto-threshold method was applied. The measured area fraction (percentage of DAB stained area per ROI) obtained when 2 images were taken, was averaged. If one tissue block featured several lesions of the same type, these values were averaged. If multiple tissue blocks from the same patient featured the same lesion types, measurements from these lesions were considered separate independent values.

Cell counts were conducted using an Olympus BX45 microscope with a U-OCMSQ 10/10 eyepiece micrometer (Olympus Lifescience) featuring a square of 10 × 10 mm2. Cells positive for CD3, CD20, IL-4 or IFNγ in lesions or NAM were counted in three random squares of 10 × 10 mm2 at 20x magnification and counts were averaged and expressed as number of positive cells/mm2.

### Isolation, culture and treatment of primary human microglia

Normal appearing human white and grey matter (5–10 g per isolation) were obtained at autopsy and stored at 4 °C in medium consisting of equal amounts of Dulbecco’s Modified Eagle Medium (DMEM; Gibco, Life Technologies, Breda, The Netherlands) and Ham’s F12 nutrient mix (Gibco, Life Technologies, Breda, The Netherlands) supplemented with 50 μg/ml gentamycin (Invitrogen, Eugene, USA). Isolation of primary microglia was conducted either directly after tissue collection or within 12 h thereafter. Subsequently, tissue was washed in collection medium and chopped using a sterile razor blade. Tissue was trypsinized for 30 min at 37 °C using 0.25% trypsin (Difco) dissolved in a trypsinization buffer (8 g/l NaCl (Sigma), 0.4 g/l KCl (Sigma, Darmstadt, Germany), 0.84 g/l NaHCO3 (Merck, Darmstadt, Germany), 0.2 g/l EDTA (Promega, Madison, USA), 4.8 g/l HEPES (Sigma), and 1 g/l glucose dissolved in MilliQ water, pH set at 7.6). After incubation, culture medium consisting of equal amounts of DMEM and Ham’s F12 supplemented with 10% fetal calf serum (Gibco, Life Technologies), 1% Penicillin/Streptomycin (Invitrogen) and 1% L-glutamine (Invitrogen) was added to de-activate the trypsin and the tissue homogenate was further dissociated using titration with a 10 ml pipette into a homogenous suspension which was filtered using a 100 μm mesh (Greiner-bio-one, Alphen aan de Rijn, The Netherlands). The suspension was centrifuged and the cell pellet was resuspended in 30% Percoll diluted in a gradient buffer (3.56 g/l of Na2HPO42H2O (Merck), 0.78 g/l of NaH2PO4H2O (Merck), 8 g/l of NaCl (Merck), 4 g/l of KCl (Merck), 2.0 g/l of d-(+)-glucose, and 2.0 g/l of BSA, pH 7.4) supplemented with 2.5% NaCl (1.5 mol/l) (GE Healthcare Biosciences AB, Uppsala, Sweden). The suspended cells were subsequently overlaid with the aforementioned gradient buffer and centrifuged for 35 min at 450×g at 18 °C with no acceleration or brake. After centrifugation, a myelin layer was formed at the interphase and microglial cells are pelleted. This cell pellet was treated with erythrocyte shock buffer (8.3 g/l of NH4Cl (Merck) and 1 g/l of KHCO3 (Merck), pH 7.4) for 15 min at 4 °C. Subsequently, cells were centrifuged and the pellet resuspended in 7.2 ml culture medium as described above and 600 μl cell suspension/well was added to a 24 well plate coated with 15 μg/mL Poly-L-Lysine (Sigma). After one day of culturing, cells were cultured in culture medium + 25 ng/ml human recombinant granulocyte-macrophage colony-stimulating factor (GM-CSF; Peprotech, London, the UK). Medium was changed every three days by replacing half of the medium with culture medium. After 7–10 days of culturing, cells were treated with recombinant human interleukin (IL)-4 (10 ng/ml; Biolegend, San Diego, USA) for 48 h or with recombinant human interferon (IFN)γ (10 ng/ml; Biolegend) for 24 h followed by addition of 10 ng/ml lipopolysaccharide (LPS, derived from E.coli O55:B5; Difco) for 24 h [33] or left untreated.

### Semi-quantitative RT-PCR

Per treatment condition, 3–5 wells containing microglial cells were lysed in a total of 1 ml TRIzol (Invitrogen). To the combined sample, 200 μl chloroform was added and tubes were centrifuged at 12,000×g for 15 min. at 4 °C. After the phenol-chloroform-extraction, RNA was purified and cleaned up using the E.Z.N.A. MicroElute RNA Clean Up kit (Omega Bio-Tek, Norcross, USA) and analyzed for quality and quantity using a NanoDrop spectrophotometer (Thermo Scientific). Input of RNA for cDNA synthesis for all samples was normalized based on the sample with the lowest concentration of RNA. Per sample, 250 ng total RNA of sufficient quality (260/230 ratio of ≥2 and 260/280 ratio ≥ 1.8) was reverse-transcribed into cDNA using the High-Capacity cDNA Reverse Transcription Kit (Applied Biosystems, Bleisswijk, The Netherlands) with oligo-d(T) primers (50 μM, Invitrogen) according to the manufacturer’s description. Semi-quantitative RT-PCR was performed in a total volume of 10 μl per sample consisting of 3 μl of Power SYBR Green Master Mix (Life Technologies, Carlsbad, USA), with 50 μM of each forward and reverse primers (see Table 3), and 6 ng/μl cDNA in a MicroAmp Optical 96-well Reaction Plate (Applied Biosystems, Foster city, USA). The PCR reaction was performed using the StepOnePlus Real-Time PCR system (Applied Biosystems). The PCR protocol was adapted from the manufacturers description and featured 40 cycles with an annealing temperature of 60 °C, followed by a melt curve analysis. The relative expression level of the target genes was determined by the LinReg PCR software (version 2014 4.3 (July 2014); website: http://www.hfrc.nl) using the following calculation N0 = Nq/ECq (N0 = target quantity, Nq = fluorescence threshold value, E = mean PCR efficiency per amplicon, Cq = threshold cycle). In total 7 housekeeping genes were tested, of which SDHA and POLR2F expression were selected for gene expression normalization using NormFinder [34]. Data analysis was performed on the normalized N0 values.

**Table 3:** Primer sequences used for qPCR

Primer	Primer sequence forward (5′- 3′)	Primer sequence reverse (3′-5′)
TMEM119	TCCAGGGTCAGATTACAAGAGCAC	ACTGTTGATTCTGGAGGGTTTGA
P2RY12	ACTCTCTCTTCCAGCCCAGGT	CCAGGACCAGTTCCTTGGCGTA
AIF-1	CCCTCCAAACTGGAAGGCTTCA	CTTTAGCTCTAGGTGAGTCTTGG
GFAP	GCAGATTCGAGAAACCAGCC	GCTCCTGCTTGGACTCCTTA
IL-1β	TACAGCTGGAGAGTGTAGATC	CAAATTCCAGCTTGTTATTG
MRC	AGTGATGGGACCCCTGTAACG	CCCAGTACCCATCCTTGCCTTT
SDHA^a^	CCAGGGAAGACTACAAGGTGCGGA	AGGGTGTGCTTCCTCCAGTGCT
POLR2F^a^	GAACTCAAGGCCCGAAAG	TGATGATGAGCTCGTCCAC

^a^ selected as housekeeping gene

### Statistical analysis

Statistical analysis was conducted using SPSS Statistics 22 (IBM, Armonk, USA). None of the semi-automatically quantified DAB stained signal datasets showed a normal distribution and were therefore analyzed using a Kruskall-Wallis test with pairwise comparisons, using the Bonferroni correction for multiple testing. *P*-values < 0.05 were considered statistically significant. Data from the semi-quantitative RT-PCR also did not show a normal distribution. In order to compare differences on a group level within WM- or GM-derived conditions, a Friedman’s test was used with post-hoc testing done manually by comparing individual data sets within WM- and GM-derived microglia with the Wilcoxon Signed Ranks test. Differences between GM and WM conditions were individually compared with the Wilcoxon Signed Ranks test, *p* values were adjusted with the Bonferroni correction. *P* values < 0.05 were considered statistically significant.

## Results

### TMEM119 and P2RY12 immunoreactivity was absent in WMLs but not in pre-active WMLs

**Fig. 2:**
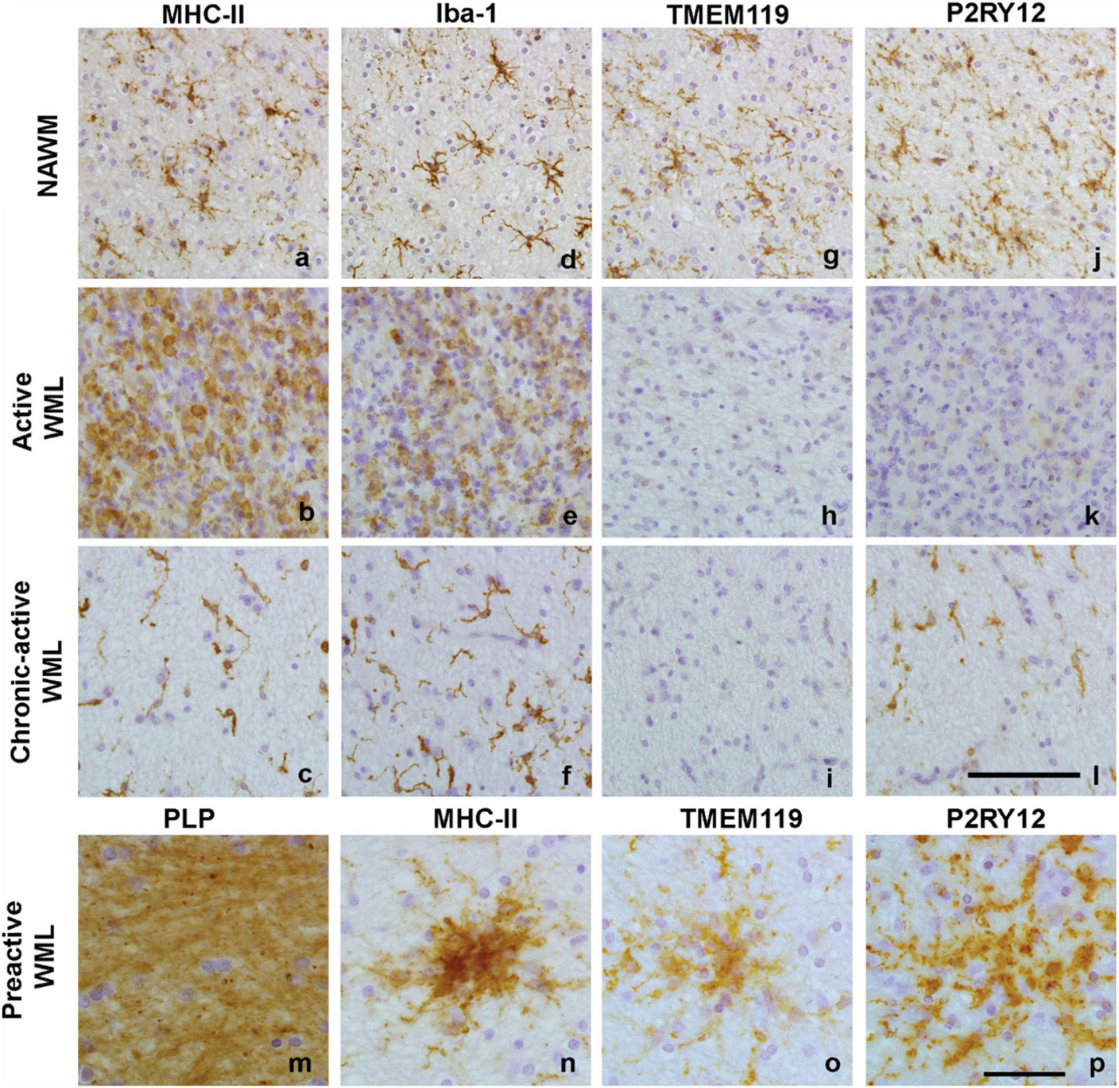
Representative images of MHC-II (**a, b, c**), Iba-1 (**d, e, f**), TMEM119 (**g, h, i**) and P2RY12 (**j, k, l**) immunoreactivity in normal appearing matter and in the demyelinated center of active WMLs and chronic-active WMLs and in pre-active lesions (**m, n, o, p**). Scalebars (**a-l** and **m-p**) = 50 μm

Compared to NAWM, where MHC-II^+^ microglia showed a ramified appearance (Fig. 2a), active WMLs showed numerous amoeboid, MHC-II^+^ cells (Fig. 2b). Chronic-active WMLs showed MHC-II^+^ cells with a more reactive phenotype (Fig. 2c). Iba-1 showed a similar pattern of immunoreactivity as MHC-II^+^ cells (Fig. 2d,e,f). In contrast, TMEM119^+^ cells were present in ramified microglia in the NAWM (Fig. 2g), but its immunoreactivity was absent in active WMLs and chronic-active WMLs (Fig. 2h, i). P2RY12^+^ cells were present in ramified cells similarly to what was observed for MHC-II^+^ and Iba-1^+^ cells in the NAWM (Fig. 2j), but they were virtually absent in active WMLs. In contrast to TMEM119, P2RY12 immunoreactivity reappeared in the center of chronic-active WMLs, showing a reactive phenotype similar to Iba-1 and MHC-II+ cells in those lesions (Fig. 2k, l). In preactive WMLs which appeared in white matter that showed no demyelination (Fig. 2m) but did show MHC-II immunoreactivity (Fig. 2n), TMEM119 (Fig. 2o) and P2RY12 (Fig. 2p) immunoreactivity was present.

### TMEM119 and P2RY12 immunoreactivity was present in subpial GMLs

**Fig. 3:**
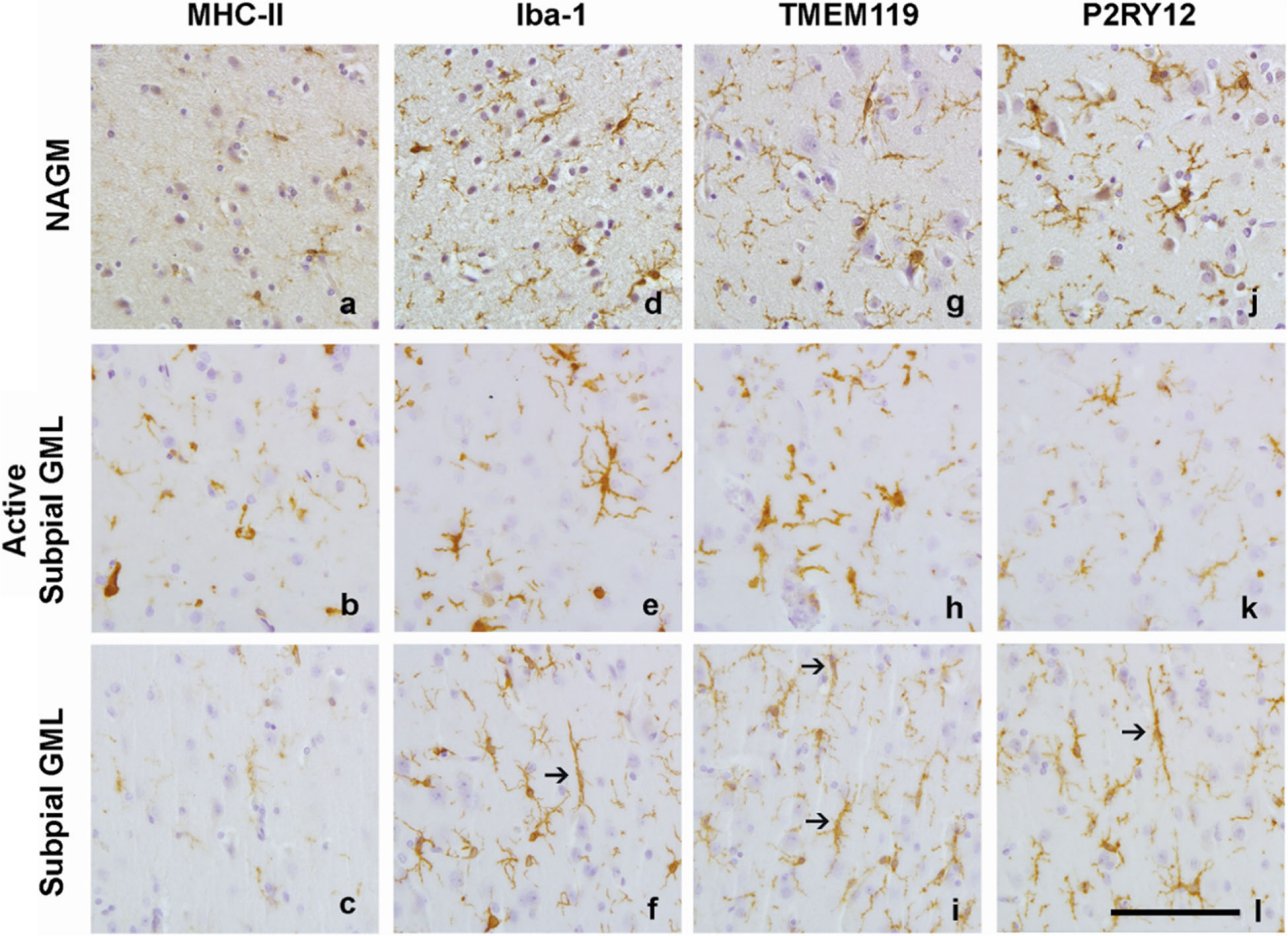
Representative images of MHC-II (**a, b, c**), Iba-1 (**d, e, f**), TMEM119 (**g, h, i**) and P2RY12 (**i, k,l**) immunoreactivity in NAGM, active subpial GMLs and non-active subpial GMLs. Arrows indicate rod-shaped microglia visible in subpial GMLs in Iba-1+ cells (**f**), TMEM119 + cells (**i**) and P2RY12+ cells (**l**). Scalebar = 50 μm

To verify that TMEM119 and P2RY12 were markers for GM microglia in addition to WM microglia, we observed that both markers completely overlap with Iba-1^+^ microglia in white- and grey normal appearing matter (Additional file 1: Figure S2). Compared to NAGM, where MHC-II immunoreactivity was present in a small amount of ramified microglia, MHC-II^+^ microglia showed a more reactive phenotype in the active subpial GML (Fig. 3a, b). Subpial GMLs without an active rim showed limited MHC-II immunoreactivity similar to NAGM (Fig. 3c). Iba-1 immunoreactivity in the NAGM was present in ramified microglia (Fig. 3d). Similar to MHC-II immunoreactivity, active subpial GML showed Iba-1 immunoreactivity in microglia with a more reactive phenotype (Fig. 3e) whereas Iba-1 immunoreactivity in less inflammatory subpial GMLs often was visible in rod-like microglia (Fig. 3f). Immunoreactivity for TMEM119 was found in  ramified microglia in the NAGM (Fig. 3g), more reactive TMEM119^+^ microglia were present in active subpial GMLs (Fig. 3h) and in rod-like microglia in subpial GMLs (Fig. 3i). P2RY12 immunoreactivity was clearly present in microglia  in NAGM (Fig. 3j), in active subpial GMLs (Fig. 3k) and in (rod-like) microglia in subpial GMLs (Fig. 3l).

### TMEM119 and P2RY12 microglial immunoreactivity is decreased in WMLs, but not in subpial GMLs and leukocortical GMLs

**Fig. 4:**
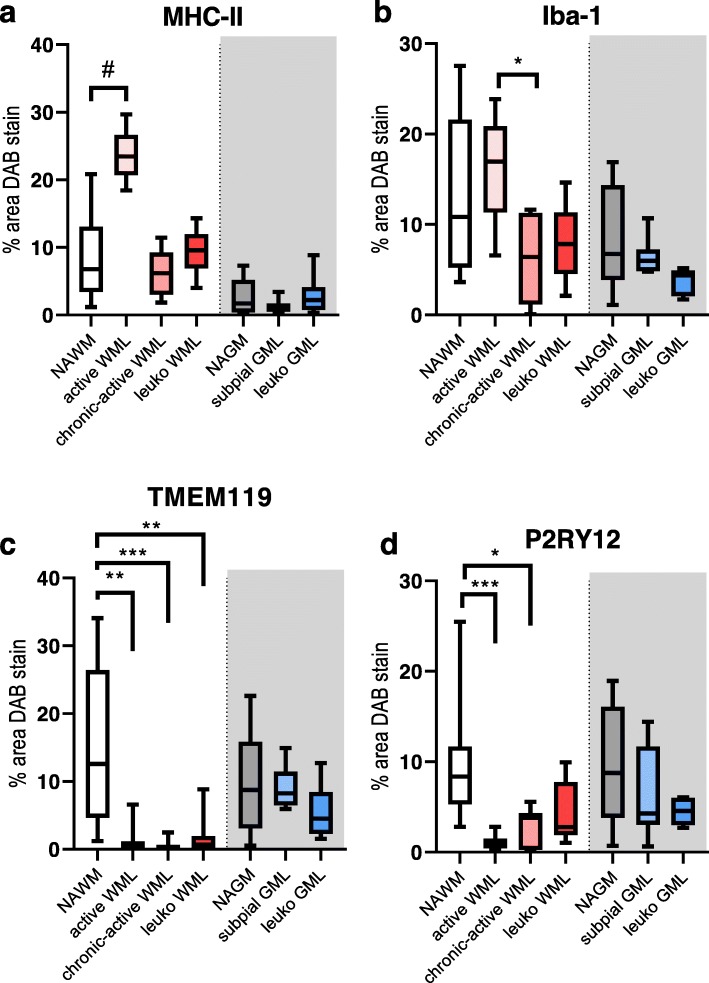
Boxplot of semi-automatic quantification of the of the DAB stained area as percentage of the ROI. in the demyelinated center of lesions compared to normal appearing matter. Boxplots represent the mean, the 1st and 4th quartile and the minimum and maximum value. Post-hoc testing was done between WM groups and between GM groups. *N* = 15 for NAWM, *N* = 10 for active WML, *N* = 7 for chronic-active WML, *N* = 5 for leuko WML, *N* = 16 for NAGM, *N* = 8 for subpial GML, *N* = 5 for leuko GML. # = *p* = 0.07, ^*^ = *p* < 0.05, ** = *p* < 0.01, *** = *p* < 0.001

Semi-automatic quantification of the DAB stained area for MHC-II, Iba-1, TMEM119 and P2RY12 was conducted on all lesion types, including leukocortical (type 1) lesions. The type 1 lesions were added to the analysis to exclude that the differences in immunoreactivity found between GMLs and WMLs were either due to location, or due to time of lesion development. Analysis of MHC-II immunoreactivity revealed a significant difference between all lesion types (Fig. 4a, X^2^(6) = 49.459, *p* < 0.01). MHC-II immunoreactivity was significant between NAWM and NAGM (*p* < 0.05) and NAWM and active WML (p < 0.05, Fig. 4a). Iba-1 immunoreactivity was also significantly different between all lesion types (Fig. 4b, X^2^(6) = 21.202, p < 0.01). Post-hoc testing revealed a significant difference in immunoreactivity between chronic-active WMLs and active WMLs (Fig. 4b, p < 0.05), likely reflecting the decrease in cell numbers observed in chronic-active WMLs compared to NAWM and active WMLs. TMEM119 immunoreactivity showed significant differences between lesion types (Fig. 4c, X^2^(6) = 42.728, *p* < 0.01). Post-hoc testing revealed a significant decrease in active WMLs (*p* < 0.01), chronic-active WMLs (*p* < 0.01) and leukocortical WMLs (*p* < 0.01) compared to NAWM. Similarly to TMEM119, P2RY12 immunoreactivity showed significant differences between lesion types (X^2^(6) = 31.705, *p* < 0.01) primarily driven by differences between NAWM and active WMLs (*p* < 0.01), and chronic active WMLs (*p* < 0.05).

### TMEM119 and P2RY12 immunoreactivity in the rim of active subpial GMLs and the rim of active WMLs appeared similar

**Fig. 5:**
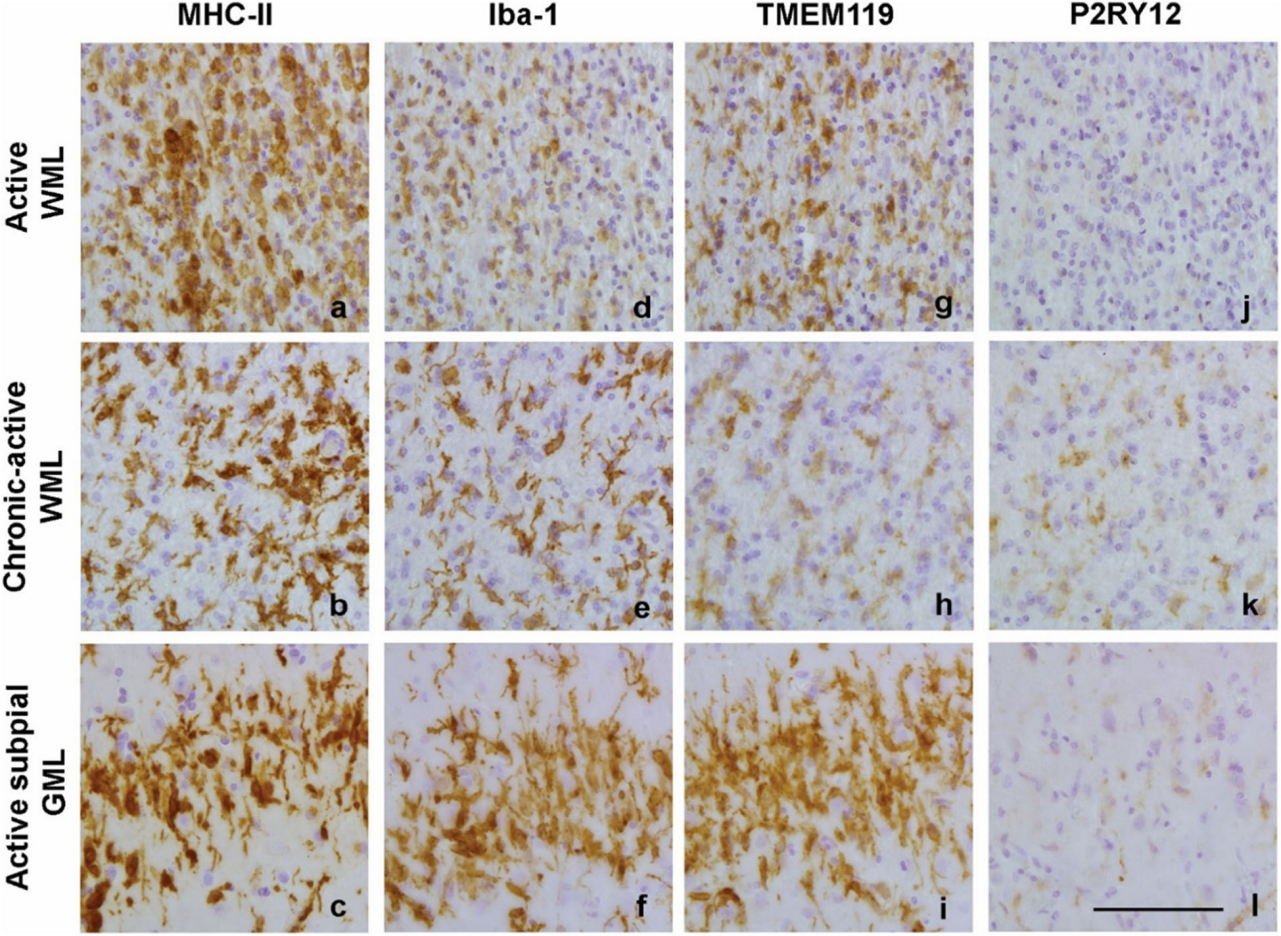
Representative images of immunoreactivity for MHC-II (**a, b, c**), Iba-1 (**d, e, f**), TMEM119 (**g, h, i**) and P2RY11 (**j, k, l**) along the rim of various lesion types. Scalebar (**a-l**) = 50 μm

MHC-II ^+^ cells and Iba1^+^ cells were present in the rim of active WMLs, chronic-active WMLs and active subpial GMLs (Fig. 5a-f). Even though immunoreactivity for TMEM119 was absent in the center of active WMLs (Fig. 2h), TMEM119 ^+^ cells were visible at the edge of active WMLs and active subpial GML, but not in the rim of chronic-active WMLs (Fig. 5g-i). P2RY12^+^ cells were absent along the rim of active WMLs but present in the rim of chronic-active WMLs (Fig. 5j-k), where immunoreactivity for P2RY12 was also visible in the center of the lesion (Fig. 2l). Similar to the edge of active WMLs, P2RY12^+^ cells were absent in the edge of active subpial GMLs (Fig. 5l).

### Regulation of TMEM119 and P2RY12 expression by pro- or anti-inflammatory mediators in primary human microglia

**Fig. 6:**
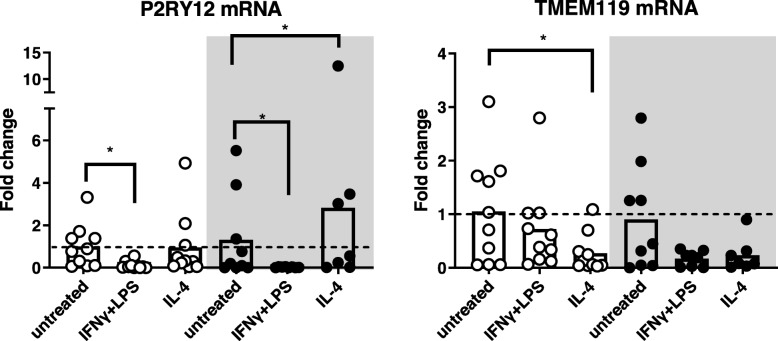
Graphs of TMEM119 and P2RY12 mRNA levels in cultured primary human microglia derived from WM enriched areas or GM enriched areas treated with IFNγ+LPS or with IL-4 compared to untreated WM microglia. mRNA levels from GM derived cells are represented in the grey-coloured box. Data are presented as individual patient-derived microglia measurements and means (bars). *N* = 10 for all WM-derived microglia conditions, *N* = 7 for IL-4 treated GM-derived microglia *N* = 8 for IFNγ+LPS treated GM-derived microglia and *N* = 9 for untreated GM-derived microglia. * = *p* < 0.05

To determine whether the differences in microglial TMEM119 and P2RY12 immunoreactivity between WMLs and GMLs were due to differences in microglial responsiveness, we isolated primary human microglia from WM (corpus callosum) and GM (cortex) tissue obtained at autopsy and treated those with IFNγ+LPS or IL-4 as representatives of a pro- or anti-inflammatory stimulus, respectively. Seven out of twelve patients from which microglia were isolated were diagnosed with MS (Table 1). The mRNA levels observed of various genes expressed in microglia of these patients did not differ from that of the five patients with other diagnoses (data not shown). The levels of TMEM119 and P2RY12 mRNA did not differ between untreated microglia derived from WM or GM. When treated with IFNγ+LPS or IL-4, primary human microglia derived from WM and GM showed upregulation of P2RY12 expression (WM: X^2^ = 10.9, df = 2, *p* < 0.01; GM: X^2^ = 12, df = 2, p < 0.01) while TMEM119 mRNA levels were only regulated in microglia derived from the WM (X2 = 7.8, df = 2, *p* < 0.05). Post-hoc testing revealed that TMEM119 mRNA was reduced after IL-4 treatment in WM-derived microglia (p < 0.05) (Fig. 6). P2RY12 mRNA level was attenuated after treatment with IFNγ+LPS in both WM- and GM-derived microglia (WM: p < 0.05, GM: p < 0.05), while after IL-4 treatment, P2RY12 expression was enhanced in the GM only (p < 0.05) (Fig. 6). However, it must be noted that variation in P2RY12 and TMEM119 mRNA levels was high in all conditions studied (see Fig. 6). Expression of AIF-1 (gene for Iba-1) did not differ between WM and GM derived microglia (Additional file 1: Figure S3). In addition our microglial cultures were not contaminated with astrocytes as shown by the lack of amplification of GFAP (Additional file 1: Figure S3). Microglia derived from both WM and GM showed downregulation of the anti-inflammatory marker mannose receptor (MRC) after treatment with IFNγ+LPS and upregulation of the pro-inflammatory marker interleukin (IL)-1β whereas treatment with IL-4 did not affect these markers (Additional file 1: Figure S3) [11, 15, 18].

### WMLs feature more infiltrated lymphocytes and lymphocyte-secreted cytokines than subpial GMLs

**Fig. 7:**
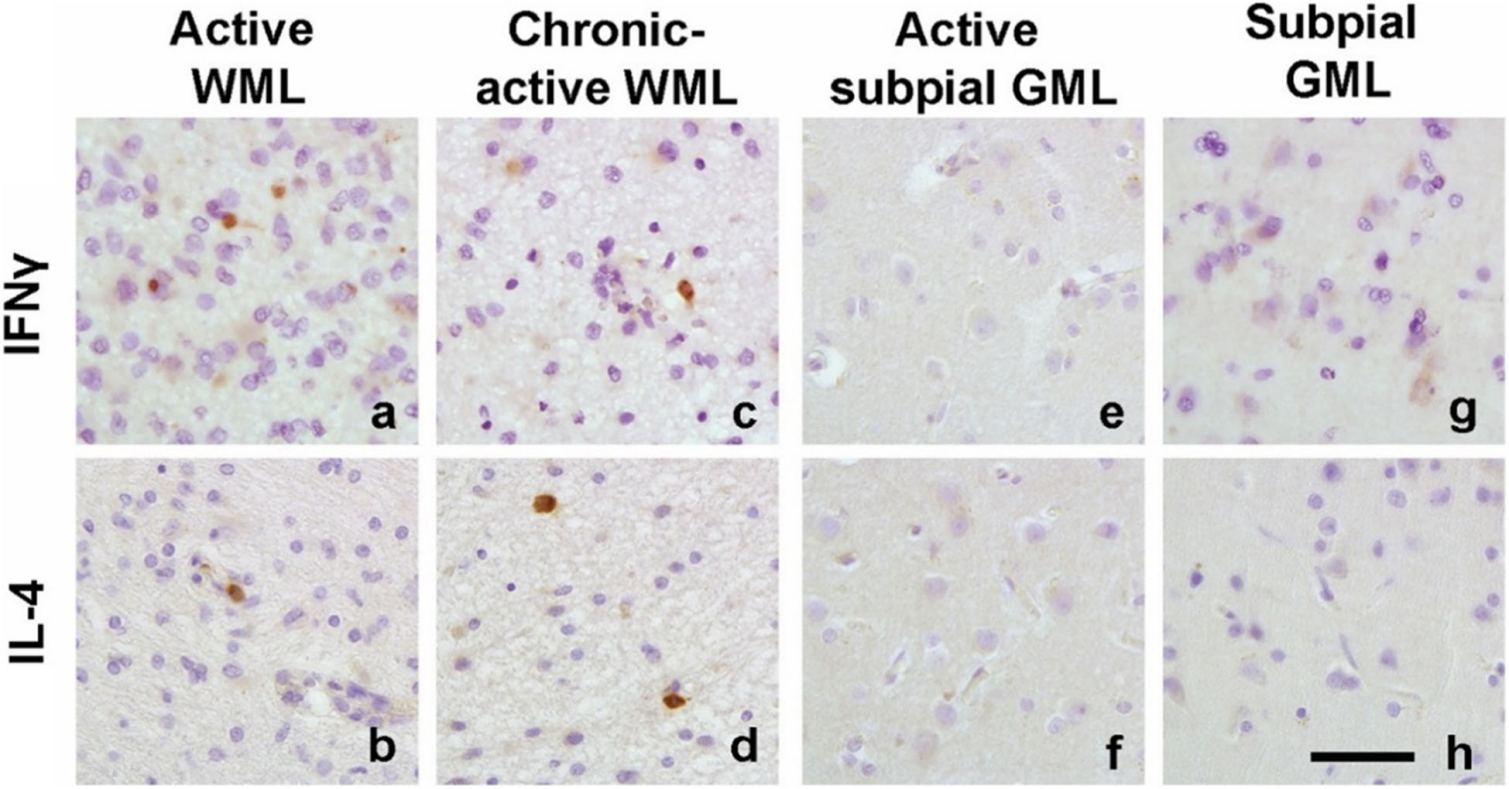
Representative images of IFNγ and IL-4 immunoreactivity in active (**a, b**) and chronic-active WMLs (**c, d**), an active subpial GML (**e, f**) and subpial GMLs (**g, h**). Scalebar (**a-h**) = 50 μm

Based on the observed regulation of TMEM119 and P2RY12 in microglia by IFNγ+LPS and IL-4, we studied the presence of lymphocytes that can produce IFNγ or IL-4 in WMLs and GMLs. A cell count of CD3^+^ (T-cells), CD20^+^ (B-cells), IL-4^+^ and IFNγ^+^ cells was conducted in WMLs, GMLs, NAWM and NAGM (Table 4). All immunohistochemical markers showed significance at the group level (CD3: X2 = 37.06, *p* < 0.0001; CD20: X2 = 11.26, *p* < 0.05; IL-4: X2 = 27.13, *p* < 0.0001; IFNγ: X2 = 21.78, *p* < 0.0002). Subsequent post-hoc analysis revealed that active WMLs had more CD3^+^ (*p* < 0.01) and IFNγ ^+^ (*p* < 0.05) cells compared to NAWM while chronic-active WMLs presented with more CD3^+^ (*p* < 0.01) and IL-4^+^cells (*p* < 0.05) (Fig. 7, Table 4). Although CD20^+^ cell counts were significantly different at the group level in the Kruskal-Wallis test pairwise comparisons, there was no significant difference between groups. When comparing immunoreactivity present in GMLs versus NAGM, no differences were found.

**Table 4:** Distribution of CD3 (+), CD20 (+), IL-4 (+) and IFNγ (+) cells/mm^2^ in MS brain tissue

	NAWM	Active WML	Chronic-active WML	NAGM	Subpial GML
CD3	2.0 ± 1.0	45.6 ± 14.5**	19.4 ± 4.2**	0.2 ± 0.1	0.7 ± 0.4
CD20	1.1 ± 0.4	4.3 ± 2.5	2.1 ± 0.6	0.4 ± 0.2	0.5 ± 0.3
IL-4	0.5 ± 0.4	1.1 ± 1.1	2.3 ± 0.6*	0.0 ± 0.0	0.0 ± 0.0
IFN-γ	0.2 ± 0.1	5.7 ± 2.8*	1.1 ± 0.5	0.0 ± 0.0	0.0 ± 0.0

Counts of CD3, CD20 and IL-4 (+) and IFNγ (+) cells in the NAWM (*N* = 18), active WMLs (*N* = 10), chronic-active WMLs (*N* = 7), NAGM (*N* = 15), subpial GMLs (*N* = 9). Data is presented as mean +/− SEM

* = *p* < 0.05, ** < *p* < 0.01

**Fig. 8:**
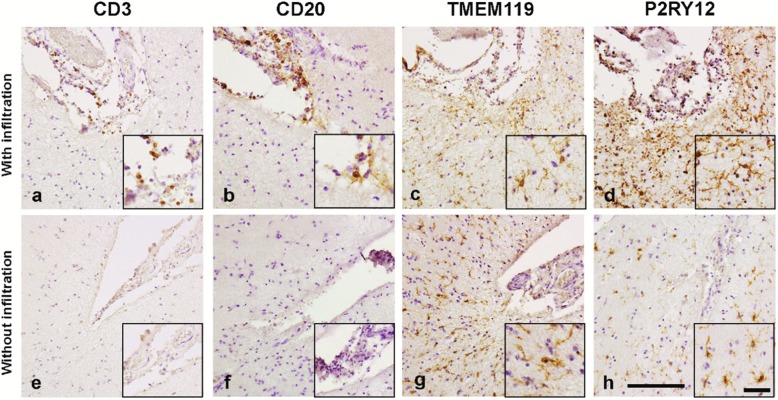
Representative images of CD3, CD20, TMEM119 and P2RY12 immunoreactivity in subpial GMLs showing meningeal infiltration and in subpial GMLs without meningeal infiltration. In subpial lesions with infiltration of CD3+ cells (**a**) and CD20+ cells (**b**) show immunoreactivity for TMEM119 (**c**) and P2RY12 (**d**). In subpial GMLs without infiltration of CD3+ (**e**) and CD20+ (**f**), immunoreactivity for TMEM119 (**g**) and P2RY12 (**h**) is similar. Scalebar (**a-h**) = 100 μm. Inserts show magnifications of the area of interest. Scalebar inserts = 50 μm

In addition, we studied whether the absence or presence of CD3^+^ and CD20^+^ cells in the meninges close to the subpial GMLs is of relevance for TMEM119 and P2RY12 immunoreactivity in subpial GMLs. We observed that TMEM119 and P2RY12 immunoreactivity in subpial GMLs was present irrespective of lymphocytes being present in meninges close to the lesions (Fig. 8).

## Discussion

The present study is the first to identify that in post-mortem material for MS patients, immunoreactivity for TMEM119 and P2RY12 in MS GMLs is different to that in WMLs. The level of TMEM119 and P2RY12 immunoreactivity hardly changes in GMLs compared to NAGM whereas clearly less immunoreactivity of both homeostatic markers was observed in WMLs compared to NAWM. Our subsequent in vitro observations of human microglia showed that TMEM119 and P2RY12 mRNA from WM and GM microglia is regulated by IFNγ+LPS and IL-4. Subsequent analysis of lymphocyte infiltration, and IFNγ and IL-4 immunoreactivity in lesions revealed lower presence of lymphocytes in GMLs than in WMLs coinciding with less IFNγ and IL-4 immunoreactivity in GMLs. We conclude that the observed difference in immunoreactivity for TMEM119 and P2RY12 in GMLs and WMLs could be due to the absence or presence of lymphocytes and inflammatory mediators in the parenchyma.

Recently, TMEM119 and P2RY12 expression in the brain is considered to represent microglia, maintaining homeostasis of the CNS [11, 12, 30]. Contrary to Iba-1 and MHC-II, TMEM119 and P2RY12 are exclusively expressed by microglia and not by infiltrated macrophages [11, 12, 35]. Therefore, in this study we utilized TMEM119 and P2RY12 expression to study microglia in WMLs and GMLs compared to normal appearing matter. Whereas we observed that in (active) WMLs, TMEM119 and P2RY12 immunoreactivity is largely absent compared to NAWM, which is in line with previous findings [16, 35], we now show that the level of TMEM119 and P2RY12 immunoreactivity is not affected in GMLs compared to NAGM. To exclude the possibility that this difference is due to distant locations of the lesions (cortical GM compared to more inflammatory WM) or due to time of development of the lesions (e.g. GML develop earlier on in the disease and are therefore less inflammatory), we verified and confirmed that in leukocortical (type 1) lesions, encompassing both WML and GML, this difference in TMEM119 and P2RY12 immunoreactivity is also present. In addition, preactive lesions in the white matter show immunoreactivity for TMEM119 and P2RY12.

Whereas in the center of active WMLs TMEM119 and P2RY12 immunoreactivity is absent, TMEM119^+^ microglia are visible surrounding the lesion, and both TMEM119^+^ and P2RY12^+^ microglia are visible in the rim of chronic-active WMLs. These findings correspond with previous observations that also showed microglial TMEM119 and P2RY12 immunoreactivity along the edge of (chronic-)active WMLs [15, 17]. Of interest is that in a subpial GML with a clear rim of MHC-II^+^ microglial cells, we observed that these microglia are TMEM119^+^ but not P2RY12^+^. This observation was similar to what was seen in the edge of active WMLs. However, immunologically active GMLs are rarely found in post-mortem MS brain material and are mostly represented by leukocortical lesions [23]. Therefore, although we cannot conclude that inflammation as seen in WMLs is present in GMLs during ongoing MS, our data suggest that the status and possible role of microglia along the edge of demyelinating lesions might be similar in active WMLs and active GMLs. In addition, we found that in subpial GMLs, rod-shaped microglia were present which were TMEM119^+^ and P2RY12^+^. Rod-shaped microglia have been proposed to play a role in synaptic stripping, representing neurodegeneration which is not necessarily mediated by inflammation [36, 37], but is present in various neurodegenerative diseases [38]. The presence of rod-shaped microglia in GMLs suggests that these cells are responsive irrespective of the relative absence of lymphocytes, and low MHC-II immunoreactivity.

We subsequently questioned whether this different expression of TMEM119 and P2RY12 of microglia in the center of GMLs versus WMLs could be explained by intrinsic differences in responsivity of WM and GM derived microglia. Indeed, P2RY12 mRNA is reduced by IFNγ+LPS in microglia from WM and GM. While studying WM-derived microglia, others have shown similar results upon IFNγ+LPS treatment, but also increased expression upon IL-4 treatment which we observed to be significantly altered in GM-derived microglia only [15, 18]. As we are not aware of any other observations on TMEM119 regulation in human microglia in vitro, we are the first to find that IL-4 treatment significantly reduced its mRNA level in WM-derived microglia. Moreover, there is a clear tendency that IFNγ+LPS reduces TMEM119 expression in microglia from both origins. Therefore, it seems that, in general, microglia derived from human WM or GM can change expression of TMEM119 or P2RY12 upon exposure to inflammatory mediators, although not entirely in a similar fashion.

Based on these in vitro observations, we next explored the possibility that the presence of IL-4 and IFNγ immunoreactivity varies between GMLs and WMLs, which would affect microglial expression of TMEM119 and P2RY12 in both lesion types. As shown in active WMLs, more IFNγ^+^ cells were found compared to the other lesion subtypes or normal-appearing matter while in chronic-active WMLs more IL-4^+^ cells were observed, but in GMLs no IL-4 or IFNγ positive cells were found. This observation is in line with our observed increased infiltration of CD3^+^ T-cells and CD20^+^ B-cells in WMLs which were relatively absent in subpial GMLs similar to as was shown before [22, 23]. Even in subpial GMLs close to meninges containing infiltrated CD3^+^ and CD20^+^ cells, we did not observe a difference in the level of immunoreactivity for TMEM119 and P2RY12. This indicates that, although recent evidence points to a role for meningeal infiltration in neuronal loss and glial activation status in MS cortex [5], microglial homeostatic status as indicated by expression of TMEM119 and P2RY12 in demyelinated subpial GM is not altered by the presence of meningeal lymphocytes and still ongoing meningeal inflammation.

The observation that P2RY12 and TMEM119 immunoreactivity is downregulated in MS WMLs and not in GMLs raises the question as to whether that has functional consequences. The ligand for P2RY12 is Adenosine diphosphate (ADP) [18] and it has been proposed that P2RY12 is involved in microglial process motility in the response of the CNS to injury [39] and upon damage to the blood-brain barrier [40]. Downregulation of P2RY12 would suggest down-tuning of microglial involvement in injury-related processes. TMEM119 was originally reported to be expressed in the plasma membrane of mouse osteoblasts and later found to be expressed in human bone tissue, dendritic cells and lymphoid tissues [16]. The presence of TMEM119 in osteosarcoma cells is related to cell invasion and migration [41], yet its function in microglia remains unknown. The recent development of microglia specific TMEM119 knock-in and CreERT2 mice [28] will be a useful tool to gain more knowledge on the functional role of TMEM119.

Thus, in conclusion, these data suggest that the continued presence of TMEM119 and P2RY12 immunoreactivity in subpial GMLs could reflect the absence of IL-4 and IFNγ and low presence of infiltrating lymphocytes in the lesion parenchyma (and not meninges) compared to WMLs. However, in subpial GMLs, where lymphocytes are absent from the lesion parenchyma and TMEM119 and P2RY12 immunoreactivity is therefore still present, TMEM119 and P2RY12 immunoreactivity is observed in rod-like microglia, showing a response of homeostatic microglia to demyelination in these lesions. Furthermore, immunoreactivity for TMEM119 and P2RY12 is observed in preactive lesions in the NAWM as well as along the edge of active WMLs and GML. Though it is plausible that differences in microglial response in WMLs and GMLs could be due to a difference in time of lesion development, analysis of TMEM119 and P2RY12 immunoreactivity in leukocortical lesions spanning both WM and GM reveal a similar pattern of immunoreactivity as WMLs and subpial GMLs. It is therefore plausible that blocking the entrance of lymphocytes into the CNS of MS patients may not interfere with all possible effects of microglia in both WMLs and GMLs.

## Supplementary information

**Additional file 1.** **Figures S1-S3**.
