# Supplementary material for: Regulation of microglial TMEM119 and P2RY12 immunoreactivity in multiple sclerosis white and grey matter lesions is dependent on their inflammatory environment
Source: Acta Neuropathol Commun. 2019 Dec 11;7:206. doi: 10.1186/s40478-019-0850-z (PMC6907356; doi:10.1186/s40478-019-0850-z)
Supplement: Supplementary file 1 — Additional file 1. Figures S1-S3. [file 40478_2019_850_MOESM1_ESM.docx]

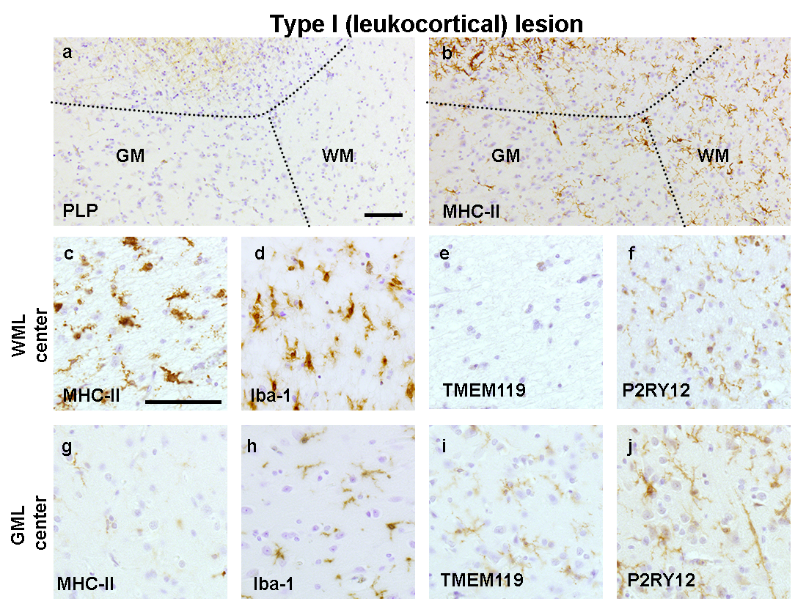


**A**

**Figure S1**: A) In type I (leukocortical) lesions, demyelination is present indicated by loss of PLP in connected WM and GM areas (a). MHC-II + cells are more abundant in the WM-part of the lesion compared to the GM-part of the lesion (b). Dashed lines indicate the edge of the lesion and the border between WM and GM. Representative images of immunoreactivity for MHC-II, Iba-1, TMEM119 ,and P2RY12 in the WM demyelinated (c, d, e, f) and GM demyelinated areas (g, h, i, j) of type I (leukocortical) lesions. Scalebars (a-b and c-j) = 50 µm. Scalebar (a,b and c-j)= 100 µm.


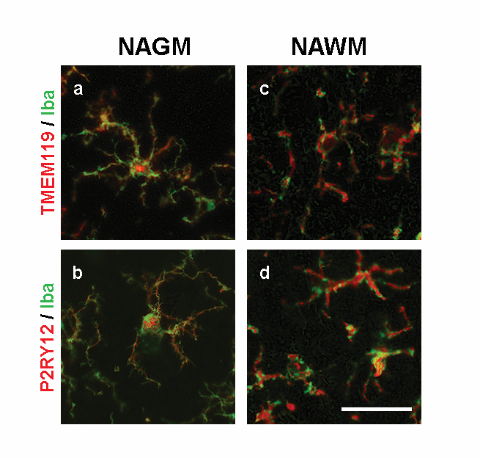


**Figure S2:** TMEM119 and P2RY12 immunoreactivity shows complete overlap with Iba-1 immunoreactivity in the normal appearing GM (a-b) and WM (c-d). Scalebar = 50 µm.

**Figure S3**: Validation of culture of primary human microglia derived from WM (left) and GM (right). WM and GM derived microglia show similar AIF-1 mRNA levels and no amplification of GFAP mRNA was foun indicating that microglial cultures were not contaminated with astrocytes (a). In addition, similar expression and regulation of MRC (b) and IL-1β mRNA (c) is found in WM and GM derived primary human microglia indicating that WM and GM derived microglia do not differ in their response to IFNγ+LPS and IL-4. Data presented as individual patient-derived microglia measurements and the mean (bars). N=10 for all WM-derived microglia conditions, N=7 for IL-4 treated GM-derived microglia N=8 for IFNγ+LPS treated GM-derived microglia and N=9 for untreated GM-derived microglia.

**a**

**b**

**c**
